# Teaching Anti-Racism in an Aging Services Course: Lessons Developed and Lessons Learned

**DOI:** 10.1093/geroni/igab046.470

**Published:** 2021-12-17

**Authors:** Althea Pestine-Stevens, Emily Greenfield

**Affiliations:** 1 Rutgers Social Work, New Brunswick, New Jersey, United States; 2 Rutgers, The State University of New Jersey, New Brunswick, New Jersey, United States

## Abstract

Despite high levels of racial disparities in health and well-being among older adults, curricula addressing how aging services systems contribute to or work to ameliorate these disparities are scarce. This paper introduces a module on inequalities and anti-racism in aging developed for an online course on aging services within a Master of Social Work program. First, materials that help students identify and understand racial inequalities in aging and in the programs that serve older adults are presented. Next, students are introduced to the applied context of how COVID-19 has exacerbated these inequalities. Finally, students critically engage in reflections and assessments of the available resources within aging services and advocacy organizations, providing recommendations for how these systems may better incorporate anti-racist practices. Challenges and opportunities will be discussed, including piloting this module in a virtual, asynchronous environment.

